# Supplementary material for: Computational Simulation of Adapter Length-Dependent LASSO Probe Capture Efficiency
Source: Biomolecules. 2019 May 22;9(5):199. doi: 10.3390/biom9050199 (PMC6571849; doi:10.3390/biom9050199)
Supplement: Supplementary file 1 [file biomolecules-09-00199-s001.zip › Supplementary_files/Table_S1_sequences_of_primers.pdf]

biomolecules

MDPI

Table 1. Sequences of primers.

0.4 um primers

Step	Name	Sequence
Fusion PCR	Blaf (Forward)	GAGTATTACCGCGGCGAATTC
	RFP200 (Rev for 200 LA	AAGCTGGAATTCGCTTCCGTACTGGAACTGAGGGC
	RFP400 (Rev for 400 LA)	AAGCTGGAATTCATGACAGGGCCATCGGAGGGG
	RFP800 (Rev for 800 LA)	AAGCTGGAATTCGAGGCGCACCGTGGGCTTG
Inversion PCR	TioINew	A*T*C*GCCGCAAGAAGTGTU
	/5Phos/SapINew	GGTTCCTGGCTCTTCGATC
	Lig_M13	GCGAACCAGACCGGAAGCA
	Ext_M13_0.6_5Phos	GATTTGGGTAATGAATATCCGGTTCTTG
	Ext_M13_1.0_5Phos	GCCGTTGCTACCCTCGTTCCGATGC
	Ext_M13_2.0_5Phos	GGCTCTGAGGGTGGCGGTTCTGAGG
	Ext_M13_4.0_5Phos	GGCGAATCCGTTATTGTTTCTCCCGATGTA
Capture PCR	PCR1kbCaptR200	CGCTTCCCTCCATCTTGACCTTAAATCTCA
	ICeuI200CaptF	CTCCCCTTCTCCTGGGATATTCTG
	PCR1kbCaptF400	GTGAAACTCAGAGGAACCAACTTCC

Both 400 & 800 nt long adaptors were amplified with PCR1kbCaptF400 primers. The "*" asterisks denote phosphorothioate bonds which protect the DNA strand from exonuclease activity.
