# Supplementary material for: Computational Simulation of Adapter Length-Dependent LASSO Probe Capture Efficiency
Source: Biomolecules. 2019 May 22;9(5):199. doi: 10.3390/biom9050199 (PMC6571849; doi:10.3390/biom9050199)
Supplement: Supplementary file 1 [file biomolecules-09-00199-s001.zip › Supplementary_files/Table_S2_sequences_of_probes.pdf]

Table S2. Sequences of probes

To clearly describe the what we have in this supplementary file, the following is a brief introduction of the construction of LASSOs. Two precursors sequences, the pre-LASSO and the long adapter sequences, are separately synthesized before assembled to form LASSO probes. Pre-LASSO could be either dsDNA oligos or ssDNA gained through oligo synthesis. It is combined by five parts, namely ligation arm, extension arm, two primer annealing sites that are going to function in the following producing procedures and one piece of sequence used for fusing the two predecessors. The long adapter is obtained from a plasmid template. It consists of one primer annealing site, the conserved linker region as well as a corresponding complementary sequence for the fusion site. After the preparation of pre-LASSO and the adapter, these two predecessors will be fused together by overlap-extension PCR. In the next step, the complex then goes through intramolecular ligation after being processed with EcoRI. Finally, the DNA circle will be transformed into a linear one with the desired sequence through inverted PCR and removal of the inversion PCR primers. For more details of construction of LASSOs, you may go through the previous publication (Tosi. et al., Nature Biomedical Engineering, 2017)

The sequences of pre-LASSOs, the long adapters as well as the final LASSO probes from inversed PCR are provided in this files. Here is a chart showing what different colors represent in the text.

GAGT….	Fusion PCR primer annealing site (Forward Primer)
GCCCTCAGTTCCAGTACGGAAGCGAATTCCAGCTT	RFP200 (Reverse Fusion PCR Primer for 200 LA)
CCCCTCCGATGGCCCTGTCATGAATTCCAGCTT	RFP400 (Reverse Fusion PCR Primer for 400 LA)
CAAGCCCACGGTGCGCCTCGAATTCCAGCTT	RFP400 (Reverse Fusion PCR Primer for 800 LA
TTGG….	Ligation arm (Sequences are inverted in the final LASSO obtained from inverted PCR)
AACA…	Inverted PCR primer annealing site (TioINew & SapINew)
GATT…	Extension arm (Sequences are inverted in the final LASSO obtained from inverted PCR)
AGAG…	Fusion overlapping sequence
ATCG…	Removed inversed PCR primer annealing sites (marked by dark green in the pre-LASSOs, corresponding to the third row of the table)
CTCC..<br>AAAC…	Primers for post-capture PCR

Individual Sequences

Pre-LASSO 0.6 kb

GAGTATTACCGCGGCGAATTCTTGGAGTTTGCTTCCGGTCTGGTTCGCAACACTTCTTGCGGCGATAGAAGGTTCCTGGCTCTTCGATCGATTTGGGTAATGAATATCCGGTTCTTGTCAAGAGAGAAGTCCTAGCACGGTAACC

Pre-LASSO 1.0 kb

GAGTATTACCGCGGCGAATTCTTGGAGTTTGCTTCCGGTCTGGTTCGCAACACTTCTTGCGGCGATAGAAGGTTCCTGGCTCTTCGATCGCCGTTGCTACCCTCGTTCCGATGCAGAGAAGTCCTAGCACGGTAACC

Pre-LASSO 1.5 & 2.0 kb

GAGTATTACCGCGGCGAATTCTTGGAGTTTGCTTCCGGTCTGGTTCGCAACACTTCTTGCGGCGATAGAAGGTTCCTGGCTCTTCGATCGGCTCTGAGGGTGGCGGTTCTGAGGAGAGAAGTCCTAGCACGGTAACC

Pre-LASSO 4.0 kb

GAGTATTACCGCGGCGAATTCTTGGAGTTTGCTTCCGGTCTGGTTCGCAACACTTCTTGCGGCGATGGTTCCTGGCTCTTCGATCGGCGAATCCGTTATTGTTTCTCCCGATGTAAGAGAAGTCCTAGCACGGTAACC

LA-242

AGAGAAGTCCTAGCACGGTAACCTCCGAGGATGTCATCAAAGAGTTTAAAGAGTTTATGAGATTTAAGGTCAAGATGGAGGGAAGCGTCAACGGACACGAGTTCGAGATTGAGGGAGAAGGAGAAGGCCGGCCTTACGAGGGCACACAAACCGCTAAGCTCAAGGTCACAAAAGGAGGACTAACTATAACGGTCCTAAGGTAGCGAACCCTCCCCTTCTCCTGGGATATTCTGAGCCCTCAGTTCCAGTACGGAAGCGAATTCCAGCTT

LA-442

AGAGAAGTCCTAGCACGGTAACCTCCGAGGATGTCATCAAAGAGTTTAAAGAGTTTATGAGATTTAAGGTCAAGATGGAGGGAAGCGTCAACGGACACGAGTTCGAGATTGAGGGAGAAGGAGAAGGCCGGCCTTACGAGGGCACACAAACCGCTAAGCTCAAGGTCACAAAAGGAGGACTAACTATAACGGTCCTAAGGTAGCGAACCCTCCCCTTCTCCTGGGATATTCTGAGCCCTCAGTTCCAGTACGGAAGCAAAGCCTATGTTAAACACCCTGCCGACATCCCTGACTATCTGAAGCTCTCCTTCCCTGAAGGCTTCAAGTGGGAGAGATTCATGAACTTCGAGGACGGAGGCGTGGTGACAGTCACACAAGATAGCACCCTCCAGGACGGAGAGTTTATTTATAAGGTGAAACTCAGAGGAACCAACTTCCCCTCCGATGGCCCTGTCATGAATTCCAGCTT

LA-750

AGAGAAGTCCTAGCACGGTAACCTCCGAGGATGTCATCAAAGAGTTTATGAGATTTAAGGTCAAGATGGAGGGAAGCGTCAACGGACACGAGTTCGAGATTGAGGGAGAAGGAGAAGGCCGGCCTTACGAGGGCACACAAACCGCTAAGCTCAAGGTCACAAAAGGAGGACTAACTATAACGGTCCTAAGGTAGCGAACCCTCCCCTTCTCCTGGGATATTCTGAGCCCTCAGTTCCAGTACGGAAGCAAAGCCTATGTTAAACACCCTGCCGACATCCCTGACTATCTGAAGCTCTCCTTCCCTGAAGGCTTCAAGTGGGAGAGATTCATGAACTTCGAGGACGGAGGCGTGGTGACAGTCACACAAGATAGCACCCTCCAGGACGGAGAGTTTATTTATAAGGTGAAACTCAGAGGAACCAACTTCCCCTCCGATGGCCCTGTCATGCAAAAAAAAACAATGGGATGGGAAGCCTCCACCGAGAGAATGTATCCTGAGGATGGCGCTCTGAAAGGCGAAATTAAAATGAGACTGAAACTCAAAGACGGAGGACACTACGATGCCGAGGTCAAAACAACCTACAAGGCCAAGAAACAAGTGCAGCTGCCTGGCGCCTACATGACTGATATTAAACTCGACATTATCAGCCATAATGGGGACTACACCATCGTGGAACAATATGAGAGAGCTGAGGGCAGACATAGCACAGGCGCTGGACAAGCCCACGGTGCGCCTCGAATTCCAGCTT

Fusion Sequences

M13_0.6-LA_0.2

Fusion

GAGTATTACCGCGGCGAATTCTTGGAGTTTGCTTCCGGTCTGGTTCGCAACACTTCTTGCGGCGATAGAAGGTTCCTGGCTCTT
CGATCGATTTGGGTAATGAATATCCGGTTCTTGTCAAGAGAGAAGTCCTAGCACGGTAACCTCCGAGGATGTCATCAAAGAGT
TTAAAGAGTTTATGAGATTTAAGGTCAAGATGGAGGGAAGCGTCAACGGACACGAGTTCGAGATTGAGGGAGAAGGAGAAG
GCCGGCCTTACGAGGGCACACAAACCGCTAAGCTCAAGGTCACAAAAGGAGGACTAACTATAACGGTCCTAAGGTAGCGAAC
CCTCCCCTTCTCCTGGGATATTCTGAGCCCTCAGTTCCAGTACGGAAGCGAATTCCAGCTT

Inv_M13_0.6-LA_0.2

ATCGCCGCAAGAAGTGTTGCGAACCAGACCGGAAGCAAACTCCAAGAATTCGCTTCCGTACTGGAACTGAGGGCTCAGAATAT
CCCAGGAGAAGGGGAGGGTTCGCTACCTTAGGACCGTTATAGTTAGTCCTCCTTTTGTGACCTTGAGCTTAGCGGTTTGTGTGC
CCTCGTAAGGCCGGCCTTCTCCTTCTCCCTCAATCTCGAACTCGTGTCCGTTGACGCTTCCCTCCATCTTGACCTTAAATCTCATA
AACTCTTTAAACTCTTTGATGACATCCTCGGAGGTTACCGTGCTAGGACTTCTCTCTTGACAAGAACCGGATATTCATTACCCAA
ATCGATCGAAGAGCCAGGAACC

GGTTCCTGGCTCTTCGATCGATTTGGGTAATGAATATCCGGTTCTTGTCAAGAGAGAAGTCCTAGCACGGTAACCTCCGAGGAT
GTCATCAAAGAGTTTAAAGAGTTTATGAGATTTAAGGTCAAGATGGAGGGAAGCGTCAACGGACACGAGTTCGAGATTGAGG
GAGAAGGAGAAGGCCGGCCTTACGAGGGCACACAAACCGCTAAGCTCAAGGTCACAAAAGGAGGACTAACTATAACGGTCC
TAAGGTAGCGAACCCTCCCCTTCTCCTGGGATATTCTGAGCCCTCAGTTCCAGTACGGAAGCGAATTCTTGGAGTTTGCTTCCG
GTCTGGTTCGCAACACTTCTTGCGGCGAT(reverse complement of the sequence above)

Inv_M13_0.6-LA_0.4

ATCGCCGCAAGAAGTGTTGCGAACCAGACCGGAAGCAAACTCCAAGAATTCATGACAGGGCCATCGGAGGGGAAGTTGGTTC
CTCTGAGTTTCACCTTATAAATAAACTCTCCGTCCTGGAGGGTGCTATCTTGTGTGACTGTCACCACGCCTCCGTCCTCGAAGTT
CATGAATCTCTCCCACTTGAAGCCTTCAGGGAAGGAGAGCTTCAGATAGTCAGGGATGTCGGCAGGGTGTTTAACATAGGCTT
TGCTTCCGTACTGGAACTGAGGGCTCAGAATATCCCAGGAGAAGGGGAGGGTTCGCTACCTTAGGACCGTTATAGTTAGTCCT
CCTTTTGTGACCTTGAGCTTAGCGGTTTGTGTGCCCTCGTAAGGCCGGCCTTCTCCTTCTCCCTCAATCTCGAACTCGTGTCCGTT
GACGCTTCCCTCCATCTTGACCTTAAATCTCATAAACTCTTTAAACTCTTTGATGACATCCTCGGAGGTTACCGTGCTAGGACTTC
TCTCTTGACAAGAACCGGATATTCATTACCCAAATCGATCGAAGAGCCAGGAACC

Inv_M13_0.6-LA_0.8

ATCGCCGCAAGAAGTGTTGCGAACCAGACCGGAAGCAAACTCCAAGAATTCGAGGCGCACCGTGGGCTTGTCCAGCGCCTGT
GCTATGTCTGCCCTCAGCTCTCTCATATTGTTCCACGATGGTGTAGTCCCCATTATGGCTGATAATGTCGAGTTTAATATCAGTC
ATGTAGGCGCCAGGCAGCTGCACTTGTTTCTTGGCCTTGTAGGTTGTTTTGACCTCGGCATCGTAGTGTCCTCCGTCTTTGAGTT
TCAGTCTCATTTTAATTTCGCCTTTCAGAGCGCCATCCTCAGGATACATTCTCTCGGTGGAGGCTTCCCATCCCATTGTTTTTTTTT
GCATGACAGGGCCATCGGAGGGGAAGTTGGTTCCTCTGAGTTTCACCTTATAAATAAACTCTCCGTCCTGGAGGGTGCTATCTT
GTGTGACTGTCACCACGCCTCCGTCCTCGAAGTTCATGAATCTCTCCCACTTGAAGCCTTCAGGGAAGGAGAGCTTCAGATAGT
CAGGGATGTCGGCAGGGTGTTTAACATAGGCTTTGCTTCCGTACTGGAACTGAGGGCTCAGAATATCCCAGGAGAAGGGGAG
GGTTCGCTACCTTAGGACCGTTATAGTTAGTCCTCCTTTTGTGACCTTGAGCTTAGCGGTTTGTGTGCCCTCGTAAGGCCGGCCT
TCTCCTTCTCCCTCAATCTCGAACTCGTGTCCGTTGACGCTTCCCTCCATCTTGACCTTAAATCTCATAAACTCTTTGATGACATCC
TCGGAGGTTACCGTGCTAGGACTTCTCTCTTGACAAGAACCGGATATTCATTACCCAAATCGATCGAAGAGCCAGGAACC

M13_1.0-LA_0.2

Fusion

GAGTATTACCGCGGCGAATTCTTGGAGTTTGCTTCCGGTCTGGTTCGCAACACTTCTTGCGGCGATAGAAGGTTCCTGGCTCTT
CGATCGCCGTTGCTACCCTCGTTCCGATGCAGAGAAGTCCTAGCACGGTAACCTCCGAGGATGTCATCAAAGAGTTTAAAGAG
TTTATGAGATTTAAGGTCAAGATGGAGGGAAGCGTCAACGGACACGAGTTCGAGATTGAGGGAGAAGGAGAAGGCCGGCCT
TACGAGGGCACACAAACCGCTAAGCTCAAGGTCACAAAAGGAGGACTAACTATAACGGTCCTAAGGTAGCGAACCCTCCCCTT
CTCCTGGGATATTCTGAGCCCTCAGTTCCAGTACGGAAGCGAATTCCAGCTT

Inv_M13_1.0-LA_0.2

ATCGCCGCAAGAAGTGTTGCGAACCAGACCGGAAGCAAACTCCAAGAATTCGCTTCCGTACTGGAACTGAGGGCTCAGAATAT
CCCAGGAGAAGGGGAGGGTTCGCTACCTTAGGACCGTTATAGTTAGTCCTCCTTTTGTGACCTTGAGCTTAGCGGTTTGTGTGC
CCTCGTAAGGCCGGCCTTCTCCTTCTCCCTCAATCTCGAACTCGTGTCCGTTGACGCTTCCCTCCATCTTGACCTTAAATCTCATA
AACTCTTTAAACTCTTTGATGACATCCTCGGAGGTTACCGTGCTAGGACTTCTCTGCATCGGAACGAGGGTAGCAACGGCGATC
GAAGAGCCAGGAACC

Inv_M13_1.0-LA_0.4

ATCGCCGCAAGAAGTGTTGCGAACCAGACCGGAAGCAAACTCCAAGAATTCATGACAGGGCCATCGGAGGGGAAGTTGGTTC
CTCTGAGTTTCACCTTATAAATAAACTCTCCGTCCTGGAGGGTGCTATCTTGTGTGACTGTCACCACGCCTCCGTCCTCGAAGTT
CATGAATCTCTCCCACTTGAAGCCTTCAGGGAAGGAGAGCTTCAGATAGTCAGGGATGTCGGCAGGGTGTTTAACATAGGCTT
TGCTTCCGTACTGGAACTGAGGGCTCAGAATATCCCAGGAGAAGGGGAGGGTTCGCTACCTTAGGACCGTTATAGTTAGTCCT
CCTTTTGTGACCTTGAGCTTAGCGGTTTGTGTGCCCTCGTAAGGCCGGCCTTCTCCTTCTCCCTCAATCTCGAACTCGTGTCCGTT
GACGCTTCCCTCCATCTTGACCTTAAATCTCATAAACTCTTTAAACTCTTTGATGACATCCTCGGAGGTTACCGTGCTAGGACTTC
TCTGCATCGGAACGAGGGTAGCAACGGCGATCGAAGAGCCAGGAACC

Inv_M13_1.0-LA_0.8

ATCGCCGCAAGAAGTGTTGCGAACCAGACCGGAAGCAAACTCCAAGAATTCGAGGCGCACCGTGGGCTTGTCCAGCGCCTGT
GCTATGTCTGCCCTCAGCTCTCTCATATTGTTCCACGATGGTGTAGTCCCCATTATGGCTGATAATGTCGAGTTTAATATCAGTC
ATGTAGGCGCCAGGCAGCTGCACTTGTTTCTTGGCCTTGTAGGTTGTTTTGACCTCGGCATCGTAGTGTCCTCCGTCTTTGAGTT
TCAGTCTCATTTTAATTTCGCCTTTCAGAGCGCCATCCTCAGGATACATTCTCTCGGTGGAGGCTTCCCATCCCATTGTTTTTTTT
GCATGACAGGGCCATCGGAGGGGAAGTTGGTTCCTCTGAGTTTCACCTTATAAATAAACTCTCCGTCCTGGAGGGTGCTATCTT
GTGTGACTGTCACCACGCCTCCGTCCTCGAAGTTCATGAATCTCTCCCACTTGAAGCCTTCAGGGAAGGAGAGCTTCAGATAGT
CAGGGATGTCGGCAGGGTGTTTAACATAGGCTTTGCTTCCGTACTGGAACTGAGGGCTCAGAATATCCCAGGAGAAGGGGAG
GGTTCGCTACCTTAGGACCGTTATAGTTAGTCCTCCTTTTGTGACCTTGAGCTTAGCGGTTTGTGTGCCCTCGTAAGGCCGGCCT
TCTCCTTCTCCCTCAATCTCGAACTCGTGTCCGTTGACGCTTCCCTCCATCTTGACCTTAAATCTCATAAACTCTTTGATGACATCC
TCGGAGGTTACCGTGCTAGGACTTCTCTGCATCGGAACGAGGGTAGCAACGGCGATCGAAGAGCCAGGAACC

M13_2.0-LA_0.2

Fusion-LA_0.2

GAGTATTACCGCGGCGAATTCTTGGAGTTTGCTTCCGGTCTGGTTCGCAACACTTCTTGCGGCGATAGAAGGTTCCTGGCTCTT
CGATCGGCTCTGAGGGTGGCGGTTCTGAGGAGAGAAGTCCTAGCACGGTAACCTCCGAGGATGTCATCAAAGAGTTTAAAGA
GTTTATGAGATTTAAGGTCAAGATGGAGGGAAGCGTCAACGGACACGAGTTCGAGATTGAGGGAGAAGGAGAAGGCCGGCC
TTACGAGGGCACACAAACCGCTAAGCTCAAGGTCACAAAAGGAGGACTAACTATAACGGTCCTAAGGTAGCGAACCCTCCCCT
TCTCCTGGGATATTCTGAGCCCTCAGTTCCAGTACGGAAGCGAATTCCAGCTT

Inv_ M13_2.0-LA_0.2

ATCGCCGCAAGAAGTGTTGCGAACCAGACCGGAAGCAAACTCCAAGAATTCGCTTCCGTACTGGAACTGAGGGCTCAGAATAT
CCCAGGAGAAGGGGAGGGTTCGCTACCTTAGGACCGTTATAGTTAGTCCTCCTTTTGTGACCTTGAGCTTAGCGGTTTGTGTGC
CCTCGTAAGGCCGGCCTTCTCCTTCTCCCTCAATCTCGAACTCGTGTCCGTTGACGCTTCCCTCCATCTTGACCTTAAATCTCATA
AACTCTTTAAACTCTTTGATGACATCCTCGGAGGTTACCGTGCTAGGACTTCTCTCCTCAGAACCGCCACCCTCAGAGCCGATCG
AAGAGCCAGGAACC

Inv_ M13_2.0-LA_0.4

ATCGCCGCAAGAAGTGTTGCGAACCAGACCGGAAGCAAACTCCAAGAATTCATGACAGGGCCATCGGAGGGGAAGTTGGTTC
CTCTGAGTTTCACCTTATAAATAAACTCTCCGTCCTGGAGGGTGCTATCTTGTGTGACTGTCACCACGCCTCCGTCCTCGAAGTT
CATGAATCTCTCCCACTTGAAGCCTTCAGGGAAGGAGAGCTTCAGATAGTCAGGGATGTCGGCAGGGTGTTTAACATAGGCTT
TGCTTCCGTACTGGAACTGAGGGCTCAGAATATCCCAGGAGAAGGGGAGGGTTCGCTACCTTAGGACCGTTATAGTTAGTCCT
CCTTTTGTGACCTTGAGCTTAGCGGTTTGTGTGCCCTCGTAAGGCCGGCCTTCTCCTTCTCCCTCAATCTCGAACTCGTGTCCGTT
GACGCTTCCCTCCATCTTGACCTTAAATCTCATAAACTCTTTAAACTCTTTGATGACATCCTCGGAGGTTACCGTGCTAGGACTTC
TCTCCTCAGAACCGCCACCCTCAGAGCCGATCGAAGAGCCAGGAACC

Inv_ M13_2.0-LA_0.8

ATCGCCGCAAGAAGTGTTGCGAACCAGACCGGAAGCAAACTCCAAGAATTCGAGGCGCACCGTGGGCTTGTCCAGCGCCTGT
GCTATGTCTGCCCTCAGCTCTCTCATATTGTTCCACGATGGTGTAGTCCCCATTATGGCTGATAATGTCGAGTTTAATATCAGTC
ATGTAGGCGCCAGGCAGCTGCACTTGTTTCTTGGCCTTGTAGGTTGTTTTGACCTCGGCATCGTAGTGTCCTCCGTCTTTGAGTT
TCAGTCTCATTTTAATTTCGCCTTTCAGAGCGCCATCCTCAGGATACATTCTCTCGGTGGAGGCTTCCCATCCCATTGTTTTTTTT
GCATGACAGGGCCATCGGAGGGGAAGTTGGTTCCTCTGAGTTTCACCTTATAAATAAACTCTCCGTCCTGGAGGGTGCTATCTT
GTGTGACTGTCACCACGCCTCCGTCCTCGAAGTTCATGAATCTCTCCCACTTGAAGCCTTCAGGGAAGGAGAGCTTCAGATAGT
CAGGGATGTCGGCAGGGTGTTTAACATAGGCTTTGCTTCCGTACTGGAACTGAGGGCTCAGAATATCCCAGGAGAAGGGGAG
GGTTCGCTACCTTAGGACCGTTATAGTTAGTCCTCCTTTTGTGACCTTGAGCTTAGCGGTTTGTGTGCCCTCGTAAGGCCGGCCT
TCTCCTTCTCCCTCAATCTCGAACTCGTGTCCGTTGACGCTTCCCTCCATCTTGACCTTAAATCTCATAAACTCTTTGATGACATCC
TCGGAGGTTACCGTGCTAGGACTTCTCTCCTCAGAACCGCCACCCTCAGAGCCGATCGAAGAGCCAGGAACC

M13_4.0-LA_0.2

Fusion

GAGTATTACCGCGGCGAATTCTTGGAGTTTGCTTCCGGTCTGGTTCGCAACACTTCTTGCGGCGATAGAAGGTTCCTGGCTCTT
CGATCGGCGAATCCGTTATTGTTTCTCCCGATGTAAGAGAAGTCCTAGCACGGTAACCTCCGAGGATGTCATCAAAGAGTTTAA
AGAGTTTATGAGATTTAAGGTCAAGATGGAGGGAAGCGTCAACGGACACGAGTTCGAGATTGAGGGAGAAGGAGAAGGCC
GGCCTTACGAGGGCACACAAACCGCTAAGCTCAAGGTCACAAAAGGAGGACTAACTATAACGGTCCTAAGGTAGCGAACCCT
CCCCTTCTCCTGGGATATTCTGAGCCCTCAGTTCCAGTACGGAAGCGAATTCCAGCTT

Inv_ M13_4.0-LA_0.2

ATCGCCGCAAGAAGTGTTGCGAACCAGACCGGAAGCAAACTCCAAGAATTCGCTTCCGTACTGGAACTGAGGGCTCAGAATAT
CCCAGGAGAAGGGGAGGGTTCGCTACCTTAGGACCGTTATAGTTAGTCCTCCTTTTGTGACCTTGAGCTTAGCGGTTTGTGTGC
CCTCGTAAGGCCGGCCTTCTCCTTCTCCCTCAATCTCGAACTCGTGTCCGTTGACGCTTCCCTCCATCTTGACCTTAAATCTCATA
AACTCTTTAAACTCTTTGATGACATCCTCGGAGGTTACCGTGCTAGGACTTCTCTTACATCGGGAGAAACAATAACGGATTCGC
CGATCGAAGAGCCAGGAACC

Inv_ M13_4.0-LA_0.4

ATCGCCGCAAGAAGTGTTGCGAACCAGACCGGAAGCAAACTCCAAGAATTCATGACAGGGCCATCGGAGGGGAAGTTGGTTC
CTCTGAGTTTCACCTTATAAATAAACTCTCCGTCCTGGAGGGTGCTATCTTGTGTGACTGTCACCACGCCTCCGTCCTCGAAGTT
CATGAATCTCTCCCACTTGAAGCCTTCAGGGAAGGAGAGCTTCAGATAGTCAGGGATGTCGGCAGGGTGTTTAACATAGGCTT
TGCTTCCGTACTGGAACTGAGGGCTCAGAATATCCCAGGAGAAGGGGAGGGTTCGCTACCTTAGGACCGTTATAGTTAGTCCT
CCTTTTGTGACCTTGAGCTTAGCGGTTTGTGTGCCCTCGTAAGGCCGGCCTTCTCCTTCTCCCTCAATCTCGAACTCGTGTCCGTT
GACGCTTCCCTCCATCTTGACCTTAAATCTCATAAACTCTTTAAACTCTTTGATGACATCCTCGGAGGTTACCGTGCTAGGACTTC
TCTTACATCGGGAGAAACAATAACGGATTCGCCGATCGAAGAGCCAGGAACC

Inv_ M13_4.0-LA_0.8

ATCGCCGCAAGAAGTGTTGCGAACCAGACCGGAAGCAAACTCCAAGAATTCGAGGCGCACCGTGGGCTTGTCCAGCGCCTGT
GCTATGTCTGCCCTCAGCTCTCTCATATTGTTCCACGATGGTGTAGTCCCCATTATGGCTGATAATGTCGAGTTTAATATCAGTC
ATGTAGGCGCCAGGCAGCTGCACTTGTTTCTTGGCCTTGTAGGTTGTTTTGACCTCGGCATCGTAGTGTCCTCCGTCTTTGAGTT
TCAGTCTCATTTTAATTTCGCCTTTCAGAGCGCCATCCTCAGGATACATTCTCTCGGTGGAGGCTTCCCATCCCATTGTTTTTTTT
GCATGACAGGGCCATCGGAGGGGAAGTTGGTTCCTCTGAGTTTCACCTTATAAATAAACTCTCCGTCCTGGAGGGTGCTATCTT
GTGTGACTGTCACCACGCCTCCGTCCTCGAAGTTCATGAATCTCTCCCACTTGAAGCCTTCAGGGAAGGAGAGCTTCAGATAGT
CAGGGATGTCGGCAGGGTGTTTAACATAGGCTTTGCTTCCGTACTGGAACTGAGGGCTCAGAATATCCCAGGAGAAGGGGAG
GGTTCGCTACCTTAGGACCGTTATAGTTAGTCCTCCTTTTGTGACCTTGAGCTTAGCGGTTTGTGTGCCCTCGTAAGGCCGGCCT
TCTCCTTCTCCCTCAATCTCGAACTCGTGTCCGTTGACGCTTCCCTCCATCTTGACCTTAAATCTCATAAACTCTTTGATGACATCC
TCGGAGGTTACCGTGCTAGGACTTCTCTTACATCGGGAGAAACAATAACGGATTCGCCGATCGAAGAGCCAGGAACC
